# The Impacts of Air Temperature on Accidental Casualties in Beijing, China

**DOI:** 10.3390/ijerph13111073

**Published:** 2016-11-02

**Authors:** Pan Ma, Shigong Wang, Xingang Fan, Tanshi Li

**Affiliations:** 1 College of Atmospheric Science, Lanzhou University, Lanzhou 730000, Gansu, China; map14@lzu.edu.cn; 2 College of Atmospheric Science, Chengdu University of Information Technology, Chengdu 610000, Sichuan, China; 3 Department of Geography and Geology, Western Kentucky University, Bowling Green, KY 42101, USA; xingang.fan@wku.edu; 4 College of Electric Engineering, Chengdu University of Information Technology, Chengdu 610000, Sichuan, China; 5 Chinese PLA General Hospital, Beijing 100000, China; lts301@sohu.com

**Keywords:** air temperature, casualty, emergency room visits, meteorological condition, lag effect

## Abstract

Emergency room (ER) visits for accidental casualties, according to the International Classification of Deceases 10th Revision Chapters 19 and 20, include injury, poisoning, and external causes (IPEC). Annual distribution of 187,008 ER visits that took place between 2009 and 2011 in Beijing, China displayed regularity rather than random characteristics. The annual cycle from the Fourier series fitting of the number of ER visits was found to explain 63.2% of its total variance. In this study, the possible effect and regulation of meteorological conditions on these ER visits are investigated through the use of correlation analysis, as well as statistical modeling by using the Distributed Lag Non-linear Model and Generalized Additive Model. Correlation analysis indicated that meteorological variables that positively correlated with temperature have a positive relationship with the number of ER visits, and vice versa. The temperature metrics of maximum, minimum, and mean temperatures were found to have similar overall impacts, including both the direct impact on human mental/physical conditions and indirect impact on human behavior. The lag analysis indicated that the overall impacts of temperatures higher than the 50th percentile on ER visits occur immediately, whereas low temperatures show protective effects in the first few days. Accidental casualties happen more frequently on warm days when the mean temperature is higher than 14 °C than on cold days. Mean temperatures of around 26 °C result in the greatest possibility of ER visits for accidental casualties. In addition, males were found to face a higher risk of accidental casualties than females at high temperatures. Therefore, the IPEC-classified ER visits are not pure accidents; instead, they are associated closely with meteorological conditions, especially temperature.

## 1. Introduction

The increased attention to the adverse effect of meteorological conditions on human health has led to a continuous effort in exploring their intrinsic relationships. A multitude of studies have assessed the influence of weather conditions on human mortality or morbidity for chronic or infectious diseases [1,2,3,4,5]. However, few weather-health-related studies were found that have a focus on weather-related accidental casualties, which seem to happen unintentionally or from certain direct causes, such as traffic injury, assaults, or trauma. The accidental casualties of interest in this study include those classified according to Chapters 19 and 20 in the International Classification of Diseases 10th Revision (ICD-10; WHO 1994) [6]. The ICD-10 is a coding of diseases and signs, symptoms, abnormal findings, complaints, social circumstances, and external causes of injury or disease. Chapter 19 deals with injury and poisoning, and certain other consequences of external causes. Chapter 20 includes accidents, intentional self-harm, assault, events of undetermined intent, legal intervention and operations of war, and complications of medical and surgical care. To simplify the classification for this study, we have consolidated the above classification and refer to them as injury, poisoning, and external causes (IPEC).

The impact of weather on the incidence of trauma, which belongs to the above IPEC-classified casualties, was explored in some previous studies [7,8]. Bhattacharyya and Millham [9] found a highly significant relationship between maximum daily temperature and trauma admissions. Additionally, both pediatric and total trauma admissions are affected by the maximum and minimum temperatures [10]. Macgregor [11] indicated that trauma incidents occur more frequently on dry and sunny days. Stomp [12] also concluded that sunny and warm days contribute to an increased incidence of trauma. Traffic accidents and self-harming events also belong to IPEC-classified casualties, which are more likely to occur during certain weather conditions. Weather conditions not only affect the environment (e.g., road conditions), but also influence people’s physical condition as well as their mental status (e.g., emotions, reaction time, activity levels) [13,14]. It is therefore difficult to determine whether a patient’s health problems were directly caused by environmental conditions.

In addition, thermal state of environment measured by temperature determines people’s degrees of comfort and activity, as well as immunity and thermo-regulating intensity of human body. Extreme thermal states of hot and cold affects human health significantly [15,16,17]. Numerous studies regarding the association between air temperature and health outcomes for chronic or infectious diseases (mortality, morbidity, ER visits, etc.) have been reported, revealing a nonlinear relationship between health conditions and temperature ranges [18,19,20,21]. However, quantitative research on the relationship between temperature and accidental casualties is still absent and necessary.

## 2. Materials and Methods

### 2.1. Data

In order to conduct a detailed quantitative analysis on accidental casualties and temperature, we collected the details of ER visits, as classified by ICD-10 Chapters 19 and 20, from the Chinese People’s Liberation Army (PLA) General Hospital in Beijing. This included 138,740 visits classified under Chapter 19 and 48,268 classified under Chapter 20 from 1 January 2009 to 31 December 2011. Air pollution data for Beijing including daily concentrations of SO_2_, NO_2_, and PM_10_ were from the China National Environmental Monitoring Centre (http://www.cnemc.cn/). Meteorological data for Beijing for the same time period were obtained from the China Meteorological Data Sharing Service System (http://cdc.nmic.cn/home.do). Observed parameters included daily maximum and minimum temperatures, daily mean temperature, relative humidity, vapor pressure, sunshine duration, daily mean air pressure, daily temperature range, and wind speed.

### 2.2. Statistical Analysis

Linear fitting was used to extract the trend of the original time series of ER visits. Based on the capability of Fourier series [22], any periodic function or signal can be decomposed into sum of a set of sine and cosine functions. In this study, the Fourier spectral analysis was used to extract the dominant periodic information. Both of linear and nonlinear Fourier series-based fitting [23] were operated in software SPSS 18.0 (IBM, Armonk, NY, USA). With reference to the definition of warm and cold seasons in climatology, the period from April to September is the warm season in Beijing, and the rest of the months belonging to the cold season.

We employed the distributed lag nonlinear model (DLNM) [24] to assess the effects of temperature on IPEC-classified ER visits. DLNM has been used previously to describe the lagged effect of air temperature on health outcomes [16,25]. It is based on the definition of a “cross-basis”, which is a bi-dimensional space of functions describing simultaneously the shape of the relationships along both the targeted variable dimension (e.g., temperature) and the lag dimension of its occurrence [26]. The cross-basis of the daily mean, maximum, and minimum temperatures were established in this study. These effects are estimated using nonlinear smoothing functions for both dimensions, where a natural cubic spline was used for air temperature and a polynomial spline for the lag effect. In this study, the maximum lag is set to 20 days, as this is long enough to capture all temperature effects.

A generalized additive model (GAM) was used to incorporate the nonlinear effects of other meteorological elements as confounding factors, together with the “cross-basis” of temperature, to fit the relationship between weather conditions and IPEC-classified ER visits. We also conducted sensitivity analyses to choose the number of degrees of freedom (DF) for temperature and its lag. The DF was chosen so that it has the maximum explained variance and strongest significance in the GAM model. The numbers of DF for temperature and the lag effect were 4 [27] and 3, respectively. The meteorological factors were fitted using smoothing spline functions, with the number of DF selected according to Akaike’s information criterion (AIC) [28] and partial auto-correlation function (PACF) minimization of the residuals. The selected number of DF was 3 for relative humidity and sunshine duration, and 5 for wind velocity.

Relative risk (RR), which represents the risk of accidental casualties caused by a unit change of environmental conditions (e.g., high temperature), is used to quantify the impact of temperature on the number of ER visits. It is defined as the ratio of the probability of a disease development in a group exposed to the environment to the probability in a non-exposed control group [29]. RR is dimensionless and ranges from 0 to ∞, with RR = 1 meaning no connection between the exposure to an environmental condition and the disease; RR < 1 meaning that the exposure will result in a reduction of the incidence of the disease (namely, exposure is a protective factor); and RR > 1 indicates the exposure is a risk factor that increases the probability of disease occurrence.

Results of the one-sample Kolmogorov-Smirnov Test showed that the number of IPEC-classified ER visits is normally distributed. The software R (v 3.2.5) packages “dlnm” and “mgcv” (publicly available on the R comprehensive archive network (CRAN) were used for model fitting. To remove long-term fluctuations in the number of ER visits, the GAM model was adjusted for trends by including a counter variable for each day of the time-series and fitting a smoothing spline (DF = 12). Dummy variables were included in the GAM model to mark holidays. In addition, the model was adjusted for the day of week (DOW) by using a categorical dummy variable. For different time periods when Beijing has its normal or obviously reduced population, the population term is set as 0 or 1, respectively. The sunshine duration, which affected the that hours people spend outdoors, is used as a proxy to describe the influence of human behavior. The final GAM model obtained is as following:

(1) $$E\left(Y_{t}\right)=basis.T+s\left(\mathrm{trend},\mathrm{DF}=4*3\right)+\mathrm{holiday}+\mathrm{DOW}+\mathrm{population}+s\left(S,\;\mathrm{DF}=3\right)+s\left(RH,\mathrm{DF}=3\right)+s\left(V,\mathrm{DF}=5\right)+\beta_{1}SO_{2}+\beta_{2}NO_{2}+\beta_{3}PM_{10}+\alpha ,$$

where *t* refers to the day of the observation; E(*Y_t_*) denotes estimated ER admissions counted on day *t*; *basis. T* is the cross-basis of temperature metrics; s() denotes the smoothing spline functions for nonlinear variables; “trend” and “holiday” are self-explanatory; *RH, V*, and *S* represent the relative humidity, wind, and sunshine duration, respectively; $\beta_{1}\sim \beta_{3}$ are coefficients for concentrations of *SO*_2_*, NO*_2_*,* and *PM*_10_; and α is the residuals of the GAM model.

## 3. Results

### 3.1. Descriptive Statistics of Variables

The descriptive statistics of each meteorological variable and ER visit are presented in Table 1. The daily mean number of ER visits for accidental casualties is 170.78, and there are more male patients than female patients. The annual range in daily mean, minimum, and maximum temperatures (T, T_min_, and T_max_) in Beijing is −13 to 35 °C, −17 to 29 °C, and −9 to 41 °C, respectively. The daily maximum concentration of SO_2_, NO_2_, and PM_10_ are 201.64 μg/m^3^, 167.36 μg/m^3^, and 801.55 μg/m^3^, respectively.

**Figure 1:**
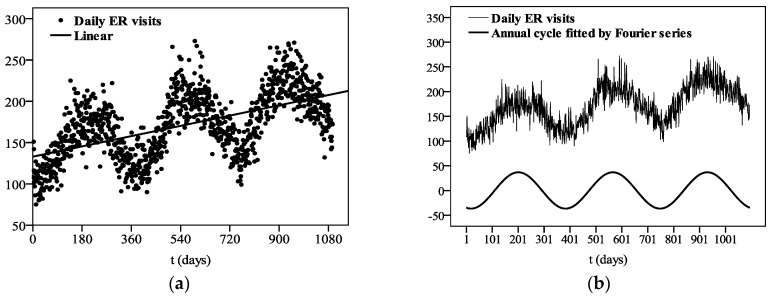
Fitted linear trend (**a**) and annual cycle (**b**) of IPEC (daily injury, poisoning, and external causes)-classified emergency room (ER) visits between 2009 and 2011 for the Chinese People’s Liberation Army (PLA) General Hospital in Beijing.

Figure 1 shows the time-series of IPEC-classified ER visits from 2009 to 2011, revealing a clear annual cycle and a sustained growth trend, with a higher number of visits in the warm seasons than in the cold seasons. Its growth trend and annual cycle were fitted by means of linear regression and Fourier series, respectively (Figure 1a,b). The linear trend can be expressed as:

(2) $$Y_{trend}=0.069t+132.90,$$

which is statistically significant at the 0.001 level, and its explained variance (R^2^) is 0.309. The annual cycle is presented as:

(3) $$Y_{cycle}=-34.86\cos\left(\frac{2\pi t}{T}\right)-11.67\sin\left(\frac{2\pi t}{T}\right),$$

where *T* refers to the length of a year (365 d), and *t* is the time variable ranging from 1 to 1095 d. In addition, the R^2^ of *Y_cycle_* is 0.632. The combination of the linear trend and annual cycle could represent most of the characteristics (R^2^ = 0.941) of the original series of ER visits. This suggests that the number of ER visits is regular to a large extent and not random nor unpredictable.

**Figure 2:**
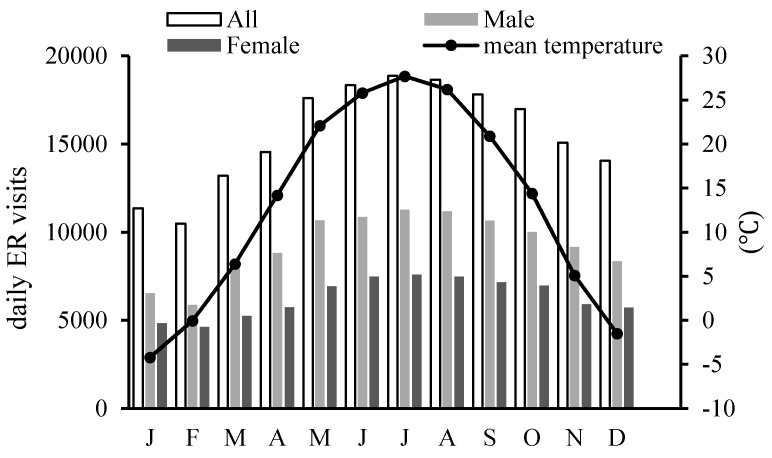
Average monthly distribution of ER visits for all patients, male, and female patients, and mean temperatures, from 2009 to 2011 in Beijing.

**Figure 3:**
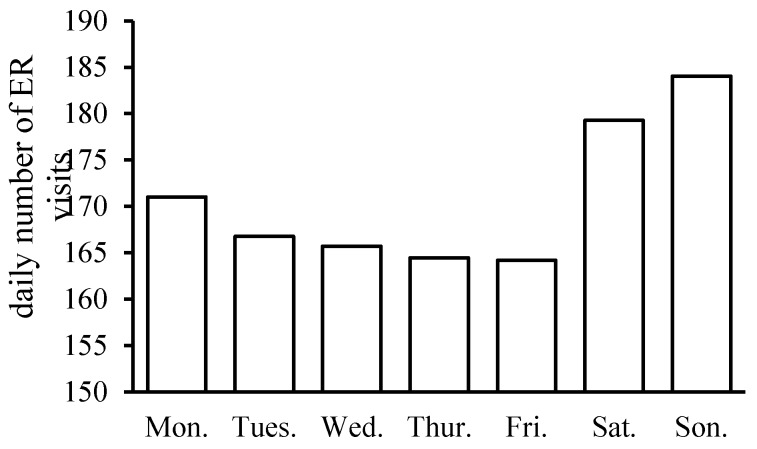
Distribution of ER visits per week from 2009 to 2011 in Beijing.

The monthly distribution of accidental casualties within a year is shown in Figure 2. The number of ER visits peaks between June and August, and is at a minimum between January and February, which is similar to the trend in the mean temperature. July witnesses the largest number of ER visits, as well as the highest monthly mean temperature, which somewhat suggests the strong effects of high temperatures on such casualties. In addition, the IPEC-classified ER visits occur discriminatively on each day of week (Figure 3). There is an evident rise in the number of visits on the two-day weekend, and there exists a sustained decreasing trend of ER visits from Monday to Friday.

### 3.2. Correlation Analysis

Table 2 lists the results of a Spearman’s correlation analysis between the number of IPEC-classified ER visits and meteorological variables. Significant positive correlations suggest that the mean/maximum/minimum temperatures, moisture conditions (relative humidity, vapor pressure), and sunshine duration are highly correlated with the number of ER visits (Table 2). The number of ER visits negatively correlates with air pressure and concentrations of SO_2_ and NO_2_. However, both air pressure and concentrations of pollutants are closely related with temperature (Table 2). Therefore, the statistical correlation is unable to explain properly all of the intrinsic links between meteorological conditions and the number of accidental casualties. However, the confounding effect of pollution was included in the GAM model in order to exclude their potential impact.

### 3.3. The Relationship between Temperature and Number of ER Visits

**Figure 4:**
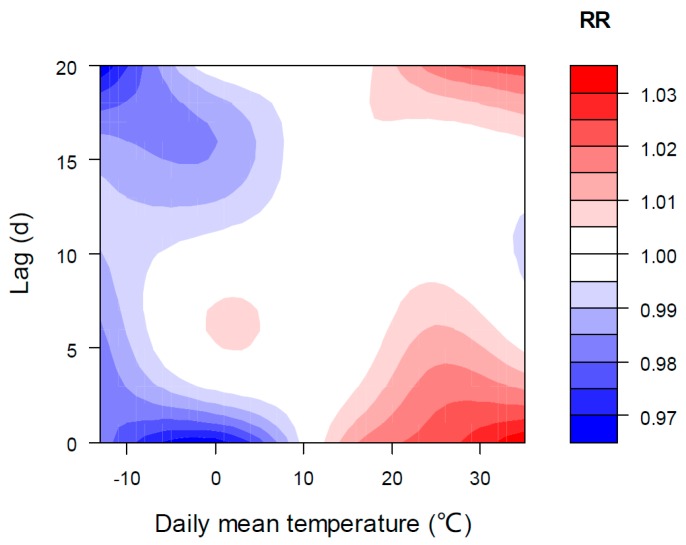
Relative risk (RR) of ER visits for mean temperature over the entire lag periods.

Temperature significantly and positively correlates with the number of ER visits and acts as a leading role among various meteorological factors. It should be noted that the results of relative risk from the time-series model only indicate the strength of relationship between the number of ER visits and temperature, which may include both direct and indirect effects of air temperature. Figure 4 shows the RR of daily mean temperature impact and its lag effect on the ER visits. It demonstrates a strong effect (high RR) of high temperatures on the first day, with a gradual reduction in RR thereafter. Low temperatures result in ER visits typically five to six days after onset, while having protective effects on the first four to five days. It should also be noted that effects of moderately high temperatures (between 20 °C and 30 °C) have a longer lag than that of extremely high temperatures. After 10 days, almost all temperature effects disappeared (Figure 4). In the long lag period (after 15 days), not much consideration should be given to the large RR centers, considering the possible over-fitting of spline functions and the large confidence intervals of RR (e.g., T = −10 °C, RR = 0.971 (95% CI: 0.939, 1.003); T = 26 °C, RR = 1.018 (95% CI: 0.996, 1.040)). 

We also explored the overall impacts of T_max_ and T_min_ on IPEC-classified ER visits and found that their results were similar to that of the mean temperature. The RR and its 95% confidence intervals of the three temperature metrics are shown in Table 3. On the day of ER visit (lag = 0), a significant increase of ER visits is revealed when the mean temperature is ≥14 °C (near its 50th percentile); otherwise, the overall effect is not statistically significant. Mean temperatures lower than 14 °C have obvious delayed influence, and the largest lag effect is when it lags 6 d. Similar to mean temperature, T_max_ and T_min_ above their 50th percentile (20.5 °C and 9.4 °C, respectively), result in an immediate increase in ER visits (Table 3). It is noted that the RR of T_min_ below its 50th percentile is not statistically significant over the entire lag period, meaning that low T_min_ has protective effects over long lag times.

**Figure 5:**
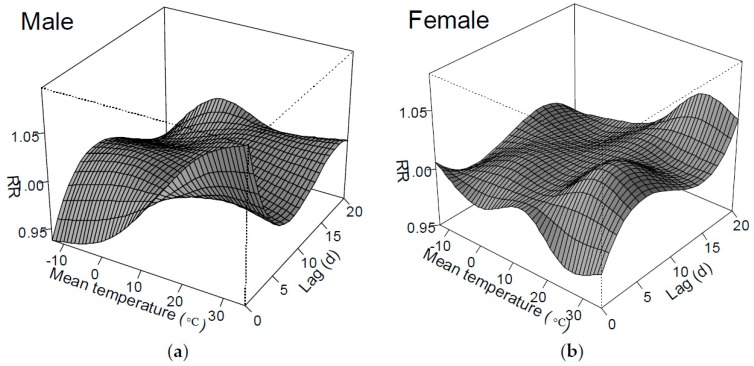
RR of different mean temperatures over the entire lag periods on ER visits of males (**a**) and females (**b**).

Figure 5 shows in three-dimensions the RR of temperature on ER visits for both males and females. For males (Figure 5a), the overall effects of high temperature seemed to be particularly evident, whereas for females, the risk seems to be more pronounced on the first couple of days in an extremely low temperature range (Figure 5b).

**Figure 6:**
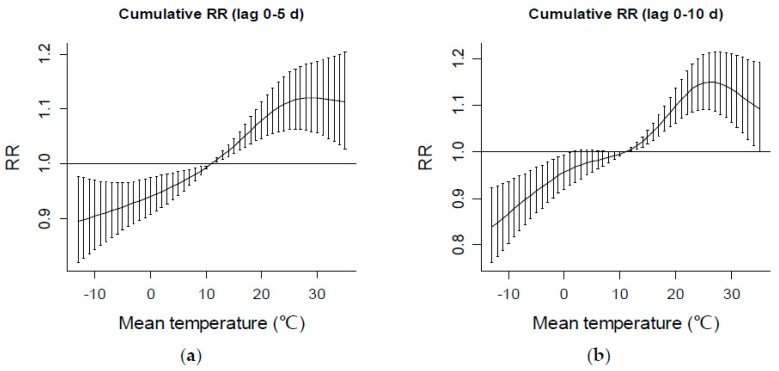
Cumulative RR of mean temperatures at two lag periods: (**a**) lag 0–5 d and (**b**) lag 0–10 d, respectively, on ER visits. Bars represent the upper and lower 95% confidence intervals of RR.

The cumulative RR of daily mean temperature for all visits at two different lag periods is presented in Figure 6. Temperatures >14 °C have significant cumulative effects (RR > 1) on ER visits, and mean temperatures around 26 °C have the greatest cumulative RR of the entire temperature range (Figure 6). Based on the cumulative RRs, there is no obvious temperature threshold with minimum effect on the daily number of ER visits. Cold temperatures show obvious protective effects. Thus, IPEC-classified accidental casualties are more likely to happen on warm or hot days than on cold days.

## 4. Discussion

This study investigated the effect of meteorological conditions on IPEC-classified ER visits. The growth trend and annual cycle of ER patients were fitted by using linear equation and Fourier series, respectively. Then, the importance and leading role of temperature was investigated, and the impacts of mean/maximum/minimum temperature on ER visits were analyzed using time-series models. Correlation analysis indicated that all meteorological variables that positively correlated with temperature have a positive relationship with ER visits, and vice versa.

The number of ER visits in February is the least throughout the year although the lowest temperature occurs in January. It could be explained by the obvious decrease of the total population in Beijing in February during the Chinese New Year celebration. It is a tradition that most of outsiders who account for a big proportion (35.9% in 2010) of permanent residents in Beijing would leave Beijing and go back to their hometowns. The rise in the number of patients on the two-day weekends could be caused by increased outdoor activities and entertainments when people are enjoying their holidays.

The time-series models considering all confounding temporal factors and nonlinear effects of meteorological variables were then established. For the purpose of eliminating the likely impacts of pollutants, the concentrations of SO_2_, NO_2_, and PM_10_ were also included in the GAM model. Previous studies on the relationship between trauma and meteorological conditions generally employed a linear correlation analysis or developed regression equations [12,30,31]. To the best of our knowledge, it is the first time that the GAM model and the more advanced DLNM model are applied in this field. Human behaviors affect the risk of injury undoubtedly, which may include doing sports, travelling, driving, laboring, etc. Unfortunately, a mediating variable that describes the number of hours that people spend outdoors was not available for this study. Within meteorological variables, the sunshine duration determines the length of time for outdoor activities to a large extent. Thus, it was also introduced into the time-series model.

A strong relationship between temperature and ER visits was revealed and analyzed, including the lag effects of daily maximum, minimum, and mean temperatures. The overall impacts of three temperature metrics are similar, including both their direct and indirect impacts. High daily mean temperatures increase the risk of accidental casualties immediately, with high numbers of visits occurring on the first day of temperature onset, followed by a gradual decline over time. At low temperature ranges, however, ER visits start to increase with a lag of five to six days.

As one aspect of the overall temperature effect, the direct impact characterizes the influence on people’s physical condition and mental status. Relevant research [32] found that significant reductions in sensory and motor amplitudes could occur in normal nerves at high temperatures. The occupants’ physiology, perceptions and mental alertness were also reported to be related to air temperature [33]. A nine-year survey found that the number of emergency psychiatric visits peaks in summer, and a strong positive association between the number of daily emergency psychiatric visits and mean daily air temperature (R = 0.82; *p* < 0.001) was shown in linear regression analysis [13]. Another study reported that fine weather conditions may increase the rate of fatal self-harm deaths, probably interacting with biological and social variables [14]. In addition, extremely high temperatures may lead to the occurrence of heat stroke, signs of heat exhaustion such as dizziness, mental confusion, headaches, and weakness, which will increase the risk of accidental casualties for them who are in high-risk occupations. Brain damage after heat stroke was also confirmed [34].

Temperature also determines people’s degrees of comfort and activity, as well as immunity and thermo-regulating intensity of human body. These are indirect impacts of temperature. In other words, temperature also affects the number of ER admissions by acting as a background or implicit factor, which affects human behaviors. If the temperature is extremely high or very low, the mechanism of thermal regulation of human body will be stimulated, and people are less likely to participate in intense activities. It implies that temperature only affects the probability of going out doors, while the direct reasons might be traffic accidents, electric shock, drowning, fires, etc.

Usually, the adverse effects of temperature, either a cold effect or a hot effect, were revealed in previous studies [2,35,36,37,38]. In the present study, only temperature that exceeds a certain threshold (14 °C) had a significant immediate effect on IPEC-classified ER visits. Therefore, 14 °C can be regarded as a critical value of mean temperature for accidental casualties. Temperatures around 26 °C were found to have the maximum cumulative RR of the entire temperature range. In Beijing, daily mean temperature around 14 °C usually occurs in April and October, and temperature ≥26 °C is common from late May to early September.

Moreover, thin clothes is a risk factor that increases body exposure to external environments, and may lead to more accidental trauma (animal bites, burns, scald, etc.).

The gender-specific effect of mean temperature was also examined. The gender differences in temperature effects on ER visits revealed that male patients are at a higher risk of accidental casualties in high temperature conditions, which may be associated with more activities (sports) that they participate in and more occupational exposure to high temperatures of men [39]. Several researchers have reported that gender differences do exist when people join outdoor physical activities [40,41,42]. Furthermore, it is known that the division of labor for males and females in a family is always distinct.

**Figure A1:**
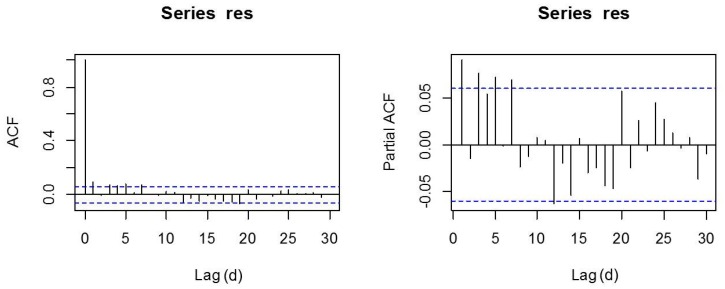
Auto-correlation (**left**) and partial auto-correlation (**right**) plots of the model residuals. Area between the blue dotted lines indicates the range of twice the standard deviation.

Residuals of the time-series auto-correlation and partial auto-correlation results from the GAM model (Figure A1) can be regarded as white noise. Although outsiders make up a big proportion of the entire population in Beijing, the probability of ER visits for accidental casualties is equal for all residents under its local weather, which is different from chronic diseases. In terms of the limitations of the data, we only analyzed ER visit data from one hospital in Beijing. Nevertheless, the relationship between IPEC-classified ER visits and air temperature was documented in Beijing under its climate background. The future studies might include data from other hospitals to increase the generalizability of the results to a larger population. This paper provides quantitative estimates of temperature impacts on IPEC-classified ER visits. The results may help to develop preventive measures and enable more efficient allocation of medical resources.

## 5. Conclusions

A strong relationship between temperature metrics and ER visits was revealed, even though the direct and indirect temperature effects were not able to be distinguished explicitly due to methodology and data limitation. Moderate to high temperatures significantly increase the risk of occurrence of accidental casualties. In addition, males face a higher risk of ER visits for accidental casualties than females under high temperature weather.

## Figures and Tables

**Table 1:** Statistics of the main meteorological variables and the number of emergency room (ER) visits for injury, poisoning, and external causes (IPEC) casualties in Beijing, from 2009 to 2011.

Variable	Mean	Standard Deviation	Variance	Minimum	Maximum	Percentile		
						25	50	75
IPEC-classified ER visits	170.78	39.34	1547.75	75	273	141	172	200
Male	101.69	24.882	619.14	38	179	83	102	120
Female	69.09	17.11	292.59	26	120	56	68	81
Air pressure (hPa)	1012.38	10.25	105.0	990	1037	1004.1	1011.8	1020.6
Temperature (°C)	13.15	11.54	133.22	−13	35	1.8	14.9	24.2
Maximum temperature (°C)	18.3	11.74	137.76	−9	41	7.5	20.5	29.1
Minimum temperature (°C)	8.45	11.40	130.0	−17	29	−2.2	9.4	19.2
Wind speed (m/s)	2.23	0.938	0.879	0	6	1.5	2.1	2.7
Sunshine duration (h)	6.74	3.99	15.89	0	14	3.6	7.7	9.8
Daily temperature range (°C)	9.85	3.58	12.78	1	22	7.3	9.7	12.1
Vapor pressure (hPa)	9.99	8.289	68.69	0	33	3.1	7	16.1
Relative humidity (%)	50.38	19.79	391.50	9	92	33	51	67
SO_2_ (μg/m^3^)	30.65	30.01	900.69	4.82	201.64	10	19.1	40.09
NO_2_ (μg/m^3^)	54.51	23.38	546.49	9	167.36	38.89	50.09	64.41
PM_10_ (μg/m^3^)	116.03	76.44	5842.4	6.4	801.55	63.86	101.91	146.95

**Table 2:** Spearman’s correlation between the number of ER patients and meteorological variables.

		Pressure (hPa)	Wind Speed (m/s)	Sunshine Duration (Hour)	Relative Humidity (%)	Mean Temperature (°C)	SO_2_ (μg/m^3^)	NO_2_ (μg/m^3^)	PM_10_ (μg/m^3^)
All	R	−0.543 **	−0.059	0.114 **	0.275 **	0.693 **	−0.528 **	−0.115 **	0.009
	P	0.000	0.051	0.000	0.000	0.000	0.000	0.000	0.764
Male	R	−0.539 **	−0.043	0.109 **	0.262 **	0.687 **	−0.501 **	−0.104 **	0.041
	P	0.000	0.151	0.000	0.000	0.000	0.000	0.001	0.180
Female	R	−0.465 **	−0.077 *	0.099 **	0.252 **	0.594 **	−0.486 **	−0.113 **	−0.035
	P	0.000	0.011	0.001	0.000	0.000	0.000	0.000	0.244
Air Pressure (hPa)	R	-	−0.048	−0.087 **	−0.334 **	−0.858 **	0.433 **	0.100 **	−0.246 **
	P	-	0.110	0.004	0.000	0.000	0.000	0.001	0.000
Wind Speed (m/s)	R	−0.048	-	0.321 **	−0.465 **	0.029	−0.158 **	−0.446 **	−0.150 **
	P	0.110	-	0.000	0.000	0.338	0.000	0.000	0.000
Sunshine Duration (h)	R	−0.087 **	0.321 **	-	−0.590 **	0.182 **	−0.278 **	−0.297 **	−0.324 **
	P	0.004	0.000	-	0.000	0.000	0.000	0.000	0.000
Daily Temperature Range (°C)	R	−0.117 **	−0.033	0.643 **	−0.368 **	0.093 **	0.028	0.122 **	0.010
	P	0.000	0.274	0.000	0.000	0.002	0.359	0.000	0.749
Vapor Pressure (hPa)	R	−0.787 **	−0.181 **	−0.138 **	0.706 **	0.900 **	−0.541 **	−0.046	0.211 **
	P	0.000	0.000	0.000	0.000	0.000	0.000	0.126	0.000
Relative Humidity (%)	R	−0.334 **	−0.465 **	−0.590 **	-	0.346 **	−0.127 **	0.268 **	0.309 **
	P	0.000	0.000	0.000	-	0.000	0.000	0.000	0.000
Mean Temperature (°C)	R	−0.858 **	0.029	0.182 **	0.346 **	-	−0.633 **	−0.201 **	0.116 **
	P	0.000	0.338	0.000	0.000	-	0.000	0.000	0.000
Maximum Temperature (°C)	R	−0.858 **	0.028	0.261 **	0.293 **	0.987 **	−0.608 **	−0.178 **	0.118 **
	P	0.000	0.356	0.000	0.000	0.000	0.000	0.000	0.000
Minimum Temperature (°C)	R	−0.836 **	0.024	0.045	0.437 **	0.979 **	−0.638 **	−0.211 **	0.129 **
	P	0.000	0.429	0.132	0.000	0.000	0.000	0.000	0.000
SO_2_ (μg/m^3^)	R	0.433 **	−0.158 **	−0.278 **	−0.127 **	−0.633 **	-	-	-
	P	0.000	0.000	0.000	0.000	0.000	-	-	-
NO_2_ (μg/m^3^)	R	0.100 **	−0.446 **	−0.297 **	0.268 **	−0.201 **	0.617 **	-	-
	P	0.001	0.000	0.000	0.000	0.000	0.000	-	-
PM_10_ (μg/m^3^)	R	−0.246 **	−0.150 **	−0.324 **	0.309 **	0.116 **	0.457 **	0.605 **	-
	P	0.000	0.000	0.000	0.000	0.000	0.000	0.000	-

R is the correlation coefficient, P is the corresponding statistical significance, ** and * indicate R is statistically significant at the 0.01 level and 0.05 level, respectively. SO_2_: Sulfur dioxide; NO_2_: Nitrogen dioxide; PM_10_: particulate matter whose particle size is less than 10 microns.

**Table 3:** Relative risk (RR) and its 95% confidence interval of all ER visits for specific percentiles of the mean, maximum, and minimum temperatures over 5 different lag periods.

**T Percentiles**	**Lag 0 d**	**Lag 2 d**	**Lag 4 d**	**Lag 6 d**	**Lag 8 d**
5th (−4.7 °C)	0.957 (0.935–0.978)	0.984 (0.975–0.993)	0.999 (0.991–1.008)	1.004 (1.001–1.013)	1.001 (0.999 –1.009)
10th (−2.9 °C)	0.956 (0.936–0.976)	0.985 (0.978–0.993)	1.001 (0.994–1.009)	1.007 (0.998–1.015)	1.004 (0.996–1.012)
25th (1.8 °C)	0.960 (0.950–0.980)	0.989 (0.985–0.996)	1.004 (0.998–1.009)	1.009 (1.002–1.015)	1.006 (1.000–1.011)
14 °C	1.003 (1.002–1.004)	1.001 (1.001–1.002)	1.000 (1.000–1.001)	1.000 (1.000–1.001)	1.000 (1.000–1.000)
50th (14.9 °C)	1.006 (1.004–1.009)	1.003 (1.002–1.004)	1.001 (1.000–1.002)	1.000 (0.999–1.002)	1.000 (0.999–1.001)
75th (24.2 °C)	1.022 (1.005–1.039)	1.021 (1.014–1.027)	1.017 (1.011–1.023)	1.013 (1.006–1.019)	1.007 (1.002–1.013)
90th (27.2 °C)	1.025 (1.006–1.045)	1.023 (1.015–1.030)	1.018 (1.012–1.025)	1.013 (1.005–1.020)	1.007 (1.000–1.013)
95th (28.7 °C)	1.026 (1.005–1.048)	1.023 (1.014–1.031)	1.017 (1.010–1.024)	1.011 (1.003–1.019)	1.005 (0.998–1.012)
**T_max_/Percentiles**	**Lag 0 d**	**Lag 2 d**	**Lag 4 d**	**Lag 6 d**	**Lag 8 d**
5th (−3 °C)	0.969 (0.939–1.001)	0.981 (0.972–0.991)	0.992 (0.980–1.004)	0.999 (0.990–1.008)	1.001 (0.992–1.009)
10th (1.76 °C)	0.966 (0.941–0.991)	0.983 (0.975–0.986)	0.997 (0.987–1.008)	1.006 (0.999–1.014)	1.008 (1.001–1.015)
25th (7.5 °C)	0.970 (0.950–0.990)	0.986 (0.970–0.993)	1.001 (0.992–1.009)	1.009 (1.002–1.015)	1.010 (1.005 –1.016)
50th (20.5 °C)	1.006 (1.003–1.009)	1.003 (1.002–1.004)	1.001 (1.000–1.002)	1.000 (0.999–1.001)	1.000 (0.999–1.001)
75th (29.1 °C)	1.030 (1.009–1.052)	1.017 (1.010–1.024)	1.012 (1.003–1.020)	1.010 (1.004–1.016)	1.009 (1.004–1.015)
90th (32.4 °C)	1.033 (1.009–1.057)	1.019 (1.011–1.027)	1.013 (1.004–1.021)	1.011 (1.004–1.012)	1.009 (1.003–1.010)
95th (34.2 °C)	1.032 (1.007–1.058)	1.019 (1.011–1.027)	1.012 (1.003–1.022)	1.010 (1.002–1.017)	1.008 (1.001–1.014)
**T_min_/Percentiles**	**Lag 0 d**	**Lag 2 d**	**Lag 4 d**	**Lag 6 d**	**Lag 8 d**
5th (−9.2 °C)	0.964 (0.933–0.996)	0.982 (0.972–0.993)	0.990 (0.977–1.004)	0.992 (0.981–1.002)	0.989 (0.979–0.999)
10th (−6.7 °C)	0.975 (0.948–1.004)	0.985 (0.968–0.994)	0.992 (0.976–0.995)	0.995 (0.978–1.000)	0.993 (0.981–1.004)
25th (−2.2 °C)	0.988 (0.966–1.011)	0.990 (0.978–0.999)	0.994 (0.982–0.998)	0.998 (0.983–1.001)	0.998 (0.985–1.004)
50th (9.4 °C)	1.001 (1.000–1.002)	1.001 (1.000–1.001)	1.000 (1.000–1.001)	1.000 (1.000–1.001)	1.000 (1.000–1.001)
75th (19.2 °C)	1.038 (1.016–1.061)	1.017 (1.014–1.036)	1.011 (1.010–1.024)	1.010 (1.005–1.020)	1.010 (1.002–1.019)
90th (23.14 °C)	1.045 (1.016–1.075)	1.016 (1.013–1.041)	1.008 (1.007–1.025)	1.008 (1.000–1.020)	1.009 (0.998–1.018)
95th (24.4 °C)	1.045 (1.014–1.077)	1.015 (1.011–1.042)	1.007 (1.005–1.025)	1.007 (0.998–1.020)	1.009 (0.996–1.018)
